# ﻿*Microbotryozyma lacustris* sp. nov. (Basidiomycota, Ustilentylomataceae) and *Cyberlindnera basumtsoensis* sp. nov. (Ascomycota, Phaffomycetaceae), two novel yeasts isolated from freshwater Lake Basom Tso, China

**DOI:** 10.3897/mycokeys.126.173807

**Published:** 2025-12-15

**Authors:** Lin Tian, Dorji Phurbu, Yan-Yan Zheng

**Affiliations:** 1 Key Laboratory of Plateau Fungi, Institute of Plateau Biology of Xizang Autonomous Region, Lhasa 850000, China Tibet Plateau Institute of Biology Lhasa China

**Keywords:** Basom Lake, *
Cyberlindnera
*, *
Microbotryozyma
*, novel species, phylogeny

## Abstract

High-altitude lakes in Tibet represent unique and underexplored reservoirs of microbial diversity. An investigation of yeast diversity in Basom Lake, a high-altitude lake in Tibet, China, led to the discovery of two novel species: *Microbotryozyma lacustris***sp. nov.** and *Cyberlindnera basumtsoensis***sp. nov.** Phylogenetic analyses of the ITS region and D1/D2 LSU rRNA gene sequences, complemented by phenotypic characterization, confirmed their distinct taxonomic status. *Microbotryozyma lacustris* represents the third species described in its genus and the first documented occurrence of *Microbotryozyma* in a freshwater habitat. *Cyberlindnera basumtsoensis* further expands the ecological diversity of the genus *Cyberlindnera*. This study significantly enriches the taxonomic framework of both genera and underscores the value of high-altitude lakes as reservoirs of novel yeast diversity.

## ﻿Introduction

High-altitude lakes in Tibet represent unique and relatively pristine ecosystems that serve as promising reservoirs for microbial diversity (Yang et al. 2022). Basom Lake (approx. 3,490 m a.s.l.), the largest glacial-dammed lake in southeastern Tibet, is characterized by its oligotrophic conditions and is surrounded by snow-capped mountains and dense forests (Wang and Dou 1998; Fang et al. 2018; Li et al. 2020a; Luo et al. 2021). Previous studies have confirmed that Tibetan aquatic systems host a high diversity of fungi, including numerous novel yeast species (Han et al. 2017; Tsuji et al. 2018; Zhou et al. 2019). A recent survey further revealed highly diverse fungal communities in Basom Lake, dominated by Ascomycota, Chytridiomycota, and Basidiomycota (Zhou et al. 2025).

The genus *Microbotryozyma* (Microbotryales) represents a relatively understudied yeast lineage. Established by Suh et al. (2012) to accommodate strains isolated from the plant bug *Collaria oleosa*, the genus initially contained only the type species *M. collariae*. The subsequent description of *M. swertiae* from the leaf surface of *Swertia yunnanensis* expanded the genus to its current two recognized species (Li et al. 2020b). While both known species were originally isolated from terrestrial sources, recent evidence suggests a broader ecological distribution. Environmental sequencing has detected *Microbotryozyma* in Lake Yamdrok (Yamzho Yumco) in Tibet, and strains of *M. collariae* have been recovered from freshwater environments in Japan (Urano et al. 2019; Hao et al. 2021). These findings indicate that *Microbotryozyma* species may inhabit both terrestrial and aquatic ecosystems (Wurzbacher et al. 2010).

The genus *Cyberlindnera* (Saccharomycetales) represents a metabolically versatile and ecologically widespread lineage (Kurtzman et al. 2008a, 2011). The genus, originally proposed as *Lindnera* before being renamed due to nomenclatural priority (Kurtzman et al. 2008b), exhibits diverse reproductive strategies and physiological capabilities (Lachance et al. 2011). Species of *Cyberlindnera* are cosmopolitan in distribution, having been isolated from diverse habitats including plant substrates, insect frass, soil, and aquatic systems (Wang et al. 2015b). This broad ecological distribution suggests important roles in carbon cycling and ecosystem functioning (Soto-Robles et al. 2019). Beyond their ecological significance, several *Cyberlindnera* species possess considerable biotechnological potential, with applications in single-cell protein production, synthesis of valuable compounds, and biofuel production from lignocellulosic biomass (Sousa-Silva et al. 2021; Bonthong et al. 2025). Recent advances in molecular systematics and genomics have further clarified the taxonomic framework of this genus (Barros et al. 2021).

Despite these advances, the yeast diversity in Basom Lake remains insufficiently explored, particularly regarding the representation of these two genera in high-altitude freshwater ecosystems. During a fungal diversity survey of this habitat, we isolated five yeast strains that could not be assigned to any known species based on preliminary sequence analysis. Phylogenetic and phenotypic characterizations confirmed that these isolates represent two novel species. Here, we formally describe these species, designated as *Microbotryozyma lacustris* sp. nov. and *Cyberlindnera basumtsoensis* sp. nov., thereby expanding the known diversity and ecological ranges of their respective genera while contributing to our understanding of yeast diversity in high-altitude aquatic environments.

## ﻿Materials and methods

### ﻿Isolation

A total of 500 mL of lake water was vacuum-filtered through a membrane using a sintered glass filter holder to capture fungal cells. Following filtration, the membrane was transferred into a sterile centrifuge tube and immediately transported to the laboratory for further processing. Serial dilutions of the fungal suspension were prepared from the membrane. From each dilution, 100 µL was spread onto yeast extract–malt extract (YM) agar plates containing 1.0% (w/v) yeast extract, 2.0% (w/v) malt extract, 0.4% (w/v) glucose, and 2.0% (w/v) agar. Chloramphenicol was added at a final concentration of 50 mg L^−1^ to inhibit bacterial growth. For each dilution, three replicate plates were prepared and incubated at 17 °C for seven days. Yeast-like colonies were selected and repeatedly streaked onto YM agar to obtain pure cultures. For long-term preservation, the purified strains were stored in glycerol suspensions at –80 °C. All type strains are maintained in a metabolically inactive state at the China General Microbiological Culture Collection Center (CGMCC) and the Japan Collection of Microorganisms (JCM).

### ﻿Phenotypic characterization

The morphological, physiological, and biochemical characteristics of the strains were assessed following standard methods (Kurtzman et al. 2011). Assimilation of carbon and nitrogen sources was examined in liquid media after subjecting the cells to a starvation period prior to inoculation. Fermentation tests were conducted using inverted Durham tubes (Chai et al. 2023). Cell morphology was observed after three days of incubation in YM broth at 17 °C by both light microscopy and scanning electron microscopy (Leica DM2500). Pseudohypha formation was evaluated on cornmeal agar (CMA; containing 2.5% cornmeal and 2% agar, w/v) with a coverslip placed over the colony to induce a semi-anaerobic environment favorable for pseudohyphal development. The sexual stage was inspected on V8 juice agar (10% V8 juice, 2% agar) and 5% malt extract agar (MEA; 5% malt extract, 1.5% agar). Growth was assessed under several conditions, covering a temperature range (17, 20, 25, 30, 35, and 37 °C), high glucose concentration (50% w/w), and vitamin-free medium. Starch production was also tested. Each strain was inoculated alone or in combination onto agar plates using a loopful of cells and incubated at 17 °C for up to two months, with periodic monitoring (Wei et al. 2024).

### ﻿Molecular phylogenetic analysis

Genomic DNA was extracted from yeast cells according to the protocol described by Kurtzman (2000). The D1/D2 domains of the large subunit (LSU) rRNA gene were amplified with primers NL1 and NL4 (Kurtzman and Robnett 1998), and the internal transcribed spacer (ITS) region was amplified using primers ITS1 and ITS4 (Schoch et al. 2012). Each PCR reaction mixture consisted of 1.0 μL of each primer (10 pM/μL), 3.0 μL of genomic DNA (10 ng/μL), and 20 μL of 1× PCR master mix (T3 Super PCR Mix, Tsingke Biotechnology Co., Ltd.). Amplification was carried out in an AB 2720 thermal cycler (Applied Biosystems, Foster City, CA, USA). PCR products were confirmed by agarose gel electrophoresis and subsequently sent to Sinogenomax (Beijing, China) for sequencing. Preliminary identification of yeast strains was conducted by BLAST searches against the GenBank database using the D1/D2 and ITS sequences as queries (Altschul et al. 1997).

Multiple sequence alignments of the ITS region and D1/D2 LSU rRNA gene domains were generated with the MAFFT program (White 1990), incorporating reference sequences obtained from GenBank (Tables 1, 2). Phylogenetic trees were reconstructed using MEGA v7.0 under the Maximum Likelihood (ML) criterion, with the best-fit substitution model selected through model testing (Wang et al. 2015a). BI analyses were conducted using a Markov Chain Monte Carlo (MCMC) algorithm in MrBayes v3.1.2 (Ronquist and Huelsenbeck 2003). Two MCMC chains were run from random trees for 1,000,000 generations, resulting in a total of 10,000 trees. The first 25% of trees sampled were discarded as the burn-in phase of each analysis. The posterior probabilities (BPP) were calculated from the remaining trees (Rannala and Yang 1996). Branch support was assessed with 1,000 bootstrap replicates (Kumar et al. 2016). *Colacogloea peniophorae* CBS 684^T^ and *Trigonopsis californica* CBS 10351^T^ were designated as outgroup taxa.

**Table 1.** Taxa used in the study of *Microbotryozyma lacustris* sp. nov. and their GenBank accession numbers.

Taxon	Strain	GenBank accessions	
		ITS	D1/D2
*Kalmanago commelinae*	SOMF 30249 ^T^	MT636665	MT636655
*Liroa emodensis*	FO 17516 ^T^	DQ238743	AY512858
*Microbotryum anomalum*	GLM 59392	EF621921	EF621960
*Microbotryum bistortarum*	TUB 015861	EF621932	EF621975
*Microbotryum bosniacum*	M-0066097	DQ238740	EF621977
*Microbotryum cordae*	B 700006023	DQ238726	EF621978
*Microbotryum dianthorum*	TUB 011802	AY588080	DQ366871
*Microbotryum holostei*	B 700006032	DQ238722	EF621981
*Microbotryum intermedium*	M 0066090	DQ238723	EF621982
*Microbotryum lychnidis-dioicae*	TUB 011796	AY588097	DQ366865
*Microbotryum marginale*	TUB 015881	EF621940	EF621989
*Microbotryum onopordi*	M-0066075	DQ238735	EF621990
*Microbotryum parlatorei*	B 700007574	DQ238736	EF621991
*Microbotryum pustulatum*	TUB015872	EF621947	EF621998
*Microbotryum reticulatum*	M-0066067	DQ238730	EF621999
*Microbotryum salviae*	GLM 50395	EF621923	EF621963
*Microbotryum saponariae*	TUB 011809	AY588089	DQ366887
*Microbotryum betonicae*	TUB 015851	EF621924	EF621964
*Microbotryum major*	B 700006042^T^	AY877419	DQ366858
*Microbotryum lychnidis-dioicae*	TUB 015865	EF621936	EF621984
*Microbotryum scabiosae*	TUB 011789	AY588083	DQ366861
*Microbotryum scorzonerae*	TUB 015878	EF621953	EF622007
*Microbotryum stygium*	M-0066047	DQ238737	EF622009
*Microbotryum tragopogonis-pratensis*	TUB 015879	EF621954	EF622014
*Microbotryum tuberculiforme*	M-0066035	DQ238744	EF622015
*Microbotryum violaceum*	TUB 011818 ^T^	AY588099	DQ366880
Sphacelotheca cf. koordersiana	JAG-55AFTOL-ID1979	DQ832221	DQ832219
*Sphacelotheca polygoni-persicariae*	KM1	MT557670	MT566306
*Sphacelotheca polygoni-serrulati*	PYCC 4293	AF444593	AF189974
*Aurantiosporium scleriae*	SOMF 30248	MT636671	MT636661
*Fulvisporium restifaciens*	DTME 306	MT636672	MT636663
*Bauerago abstrusa*	HUV 18526	DQ238719	EF621955
*Bauerago vuyckii*	MP 2380 ^T^	DQ238720	DQ363321
*Bauerozyma artemisiae*	YN 25-3 ^T^	OP470312	OP470216
***Microbotryozyma lacustris***	**CGMCC 2.8854** ^T^	**PX048001**	**PX048003**
***Microbotryozyma lacustris***	**BSC-W-3-4**	**PX499076**	**PX499117**
***Microbotryozyma lacustris***	**BSC-W-7-4**	**PX499077**	**PX499118**
*Microbotryozyma collariae*	ATCCMYA-4666 ^T^	JN849458	JN849460
*Microbotryozyma swertiae*	CGMCC 2.3533 ^T^	MK050424	MK050424
*Ustilentyloma fluitans*	RB900	AY212990	AF009882
*Ustilentyloma brefeldii*	TUB012510	DQ238745	EF622016
*Mastigobasidium intermedium*	CBS 7226	AF444564	AF189889
*Leucosporidium fellii*	CBS 7287	AF444508	AF189907
*Leucosporidiella fragaria*	CBS 6254	AF444530	AF070428
*Rhodotorula creatinovora*	CBS 8620	AF444629	AF189925
*Leucosporidium scottii*	CBS 5930 ^T^	AF444495	AY213000
*Rhodotorula mucilaginosa*	CBS 316	AF444541	AF070432
*Rhodosporidium sphaerocarpum*	CBS 5939	AF444499	AF070425
*Rhodotorula qlutinis*	CBS 20 ^T^	AF444539	AF070430
*Colacogloea peniophorae*	CBS 684 ^T^	DQ202270	AY629313

Note: Newly generated sequences are in bold. The superscript “^T^” indicates ex-type strains.

**Table 2.** Taxa used in the study of *Cyberlindnera basumtsoensis* sp. nov. and their GenBank accession numbers.

Taxon	Strain	GenBank accessions	
		ITS	D1/D2
*Candida adriatica*	ZIM 2334	HE_574654	NG_060386
***Cyberlindnera basumtsoensis***	**CGMCC 2.8853** ^T^	**PX048002**	**PX048004**
***Cyberlindnera basumtsoensis***	**Y-18-1-13-6**	**PX578846**	**PX495968**
*Cyberlindnera japonica*	NRRL YB-2750 ^T^	KY103061	EF550323
*Cyberlindnera xylosilytica*	NRRL YB-2097 ^T^	KP232976	EF550324
*Candida maesa*	ATCC MYA-4698 ^T^	HM461661	JQ812697
*Cyberlindnera veronae*	NRRL Y-7818 ^T^	AF335966	EF550322
*Cyberlindnera fabianii*	NRRL Y-1871 ^T^	AF335967	EF550321
*Candida mycetangii*	NRRL Y-6843 ^T^	KY102221	EF550330
*Candida maritima*	NRRL Y-17775 ^T^	KY102197	EF550332
*Candida nakhonratchasimensis*	JCM 12474 ^T^	KY102223	AY634567
*Cyberlindnera mississippiensis*	NRRL YB-1294 ^T^	KY103068	EF550320
*Cyberlindnera amylophila*	NRRL YB-1287 ^T^	KY103039	EF550319
*Cyberlindnera xishuangbannaensis*	NYNU 16752 ^T^	KY213821	KY213813
*Candida stauntonica*	ATCC MYA-4699 ^T^	HM461658	JQ812698
*Candida taoyuanica*	ATCC MYA-4700 ^T^	FJ873419	JQ812699
*Candida hungchunana*	ATCC MYA-4701 ^T^	HQ623543	JQ812700
*Cyberlindnera meyerae*	NRRL Y-17236 ^T^	KY103066	EF550327
*Cyberlindnera euphorbiae*	NRRL Y-17232 ^T^	KY103041	EF550326
*Cyberlindnera xylebori*	NBRC 11048 ^T^	KY103116	AB534167
*Cyberlindnera suaveolens*	NRRL Y-17391 ^T^	EU307977	EU544674
*Cyberlindnera saturnus*	NRRL Y-17396 ^T^	EU307970	EF550316
*Cyberlindnera subsufficiens*	NRRL Y-1657 ^T^	EU307975	EF550318
*Candida takata*	ATCC MYA-4702 ^T^	JQ906769	JQ906764
*Candida vartiovaarae*	NRRL Y-670 ^T^	KY102489	EF550315
*Candida mengyuniae*	CBS 10845 ^T^	EU043159	EU043158
*Cyberlindnera samutprakarnensis*	CBS 12528 ^T^	AB695388	AB598079
*Cyberlindnera jadinii*	NRRL Y-1542 ^T^	DQ249199	EF550309
*Cyberlindnera misumaiensis*	NRRL Y-17389 ^T^	KY103070	U73581
*Cyberlindnera lachancei*	NRRL Y-27008 ^T^	KY103063	EF550313
*Cyberlindnera petersonii*	NRRL YB-3808 ^T^	KY103077	EF550311
*Millerago phaffii*	IBUN-04084 ^T^	ON311286	ON264698
*Millerago galiae*	CBS 8842 ^T^	KY102096	NG058980
*Barnettozyma californica*	CBS 252 ^T^	NR138212	KY106168
*Barnettozyma hawaiiensis*	CBS 8760 ^T^	KY101728	NG058701
*Barnettozyma vustinii*	CBS 11554 ^T^	NR137724	NG058702
*Barnettozyma xylosica*	NBRC 111558 ^T^	NR154882	NG058714
*Barnettozyma populi*	CBS 8094 ^T^	NR153632	NG058630
*Barnettozyma menglunensis*	NYNU 1811121	MK682797	MK682804
*Trigonopsis californica*	CBS 10351 ^T^	KY105760	KY109968

Note: Newly generated sequences are in bold. The superscript “^T^” indicates ex-type strains.

## ﻿Results

### ﻿Phylogenetic analyses

During a survey of yeast diversity in Basom Lake (Basum Tso), Nyingchi, Tibet, China, a total of 40 water samples were collected from various depths. Among the isolates, five yeast strains exhibiting unusual phenotypic characteristics were recovered and could not be identified as any known species based on BLAST searches of the ITS and D1/D2 sequences. Subsequent phylogenetic analyses confirmed that these five strains represent two distinct novel species. Strains CGMCC 2.8854, BSC-W-3-4, and BSC-W-7-4 were assigned to the genus *Microbotryozyma*, while strains CGMCC 2.8853 and Y-18-1-13-6 were affiliated with the genus *Cyberlindnera*.

**Figure 1.**
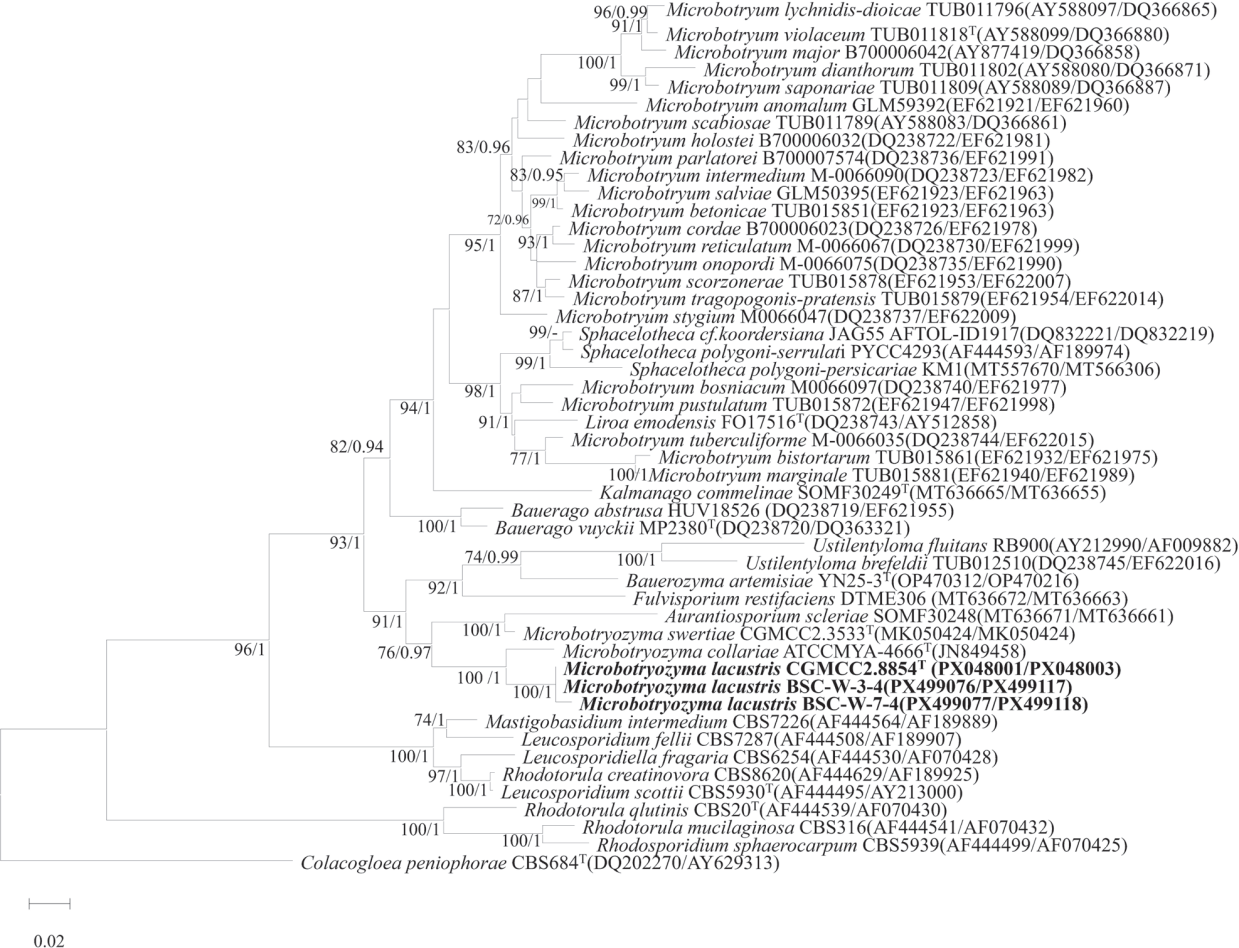
Phylogenetic tree constructed from the combined sequences of the D1/D2 and ITS regions, showing the phylogenetic positions of the type strain CGMCC 2.8854^T^ and related species. Reference strains included in the tree were either type strains of closely related species within the genus or strains widely cited in previous studies to ensure accurate phylogenetic comparison. Maximum likelihood bootstrap values (ML-BS ≥ 70%) and Bayesian posterior probabilities (BPP ≥ 0.9) are shown above the branches. *Colacogloea peniophorae* CBS 684^T^ (accession numbers: DQ202270/AY629313) was used as the outgroup. The scale bar represents a patristic distance of 0.02.

Phylogenetic analysis showed that strains CGMCC 2.8854, BSC-W-3-4, and BSC-W-7-4 formed a distinct, well-supported clade that is sister to *Microbotryozyma collariae* (type strain ATCC MYA-4666). Nucleotide comparisons with strain ATCC MYA-4666 revealed the following sequence divergences: CGMCC 2.8854 differed by 44 mismatches (29 substitutions and 15 indels, 8.78%) in the ITS region and seven substitutions (1.39%) in the D1/D2 domain; BSC-W-3-4 exhibited 35 substitutions and 17 indels in ITS, and eight substitutions and two indels in D1/D2; and BSC-W-7-4 displayed 36 substitutions and 17 indels in ITS, along with eight substitutions and two indels in D1/D2 (Fig. 1, Suppl. material 1: figs S1, S2). An unpublished strain, YYB134 (GenBank accession MT408741), also isolated from Tibet, shares identical D1/D2 sequences with CGMCC 2.8854, confirming that they represent the same taxon (Suppl. material 1: fig. S1). These results suggested that the CGMCC 2.8854 clade represents a novel species in the genus *Microbotryozyma*.

**Figure 2.**
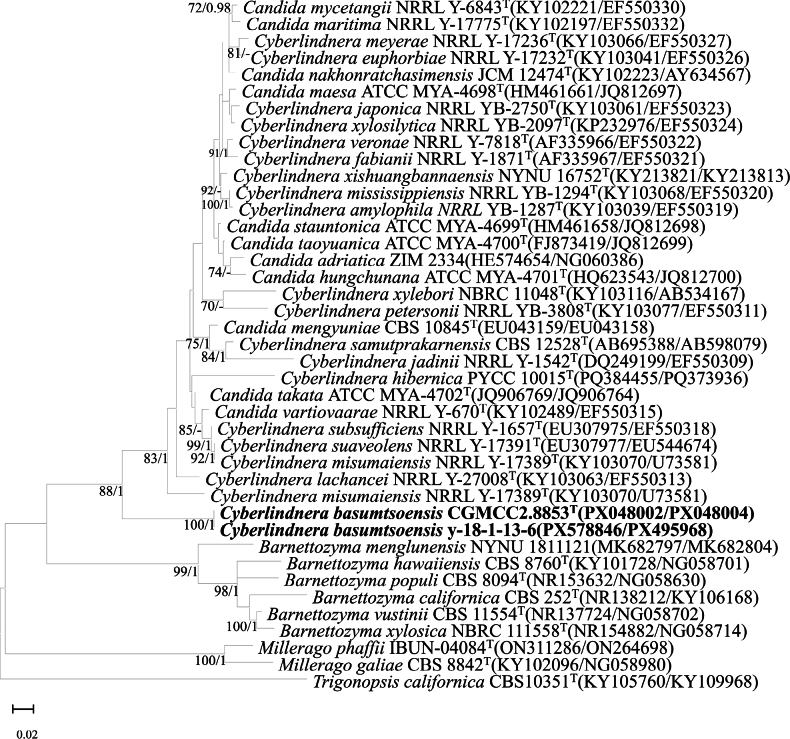
Phylogenetic tree constructed from the combined sequences of the D1/D2 and ITS regions, showing the phylogenetic positions of the type strain CGMCC 2.8853^T^ and related species. Reference strains included in the tree were either type strains of closely related species within the genus or strains widely cited in previous studies. Maximum likelihood bootstrap values (ML-BS ≥ 70%) and Bayesian posterior probabilities (BPP ≥ 0.9) are shown above the branches. *Trigonopsis californica* CBS 10351 (KY105760/KY109968) was used as the outgroup. Bar, patristic distance of 0.02.

Phylogenetic analysis showed that strains CGMCC 2.8853 and Y-18-1-13-6 formed a distinct, well-supported clade. Although this clade is located at the base of the genus in the phylogenetic tree, there is insufficient morphological and phylogenetic evidence to support its description as a novel genus; therefore, both strains are retained in the genus *Cyberlindnera* (Fig. 2, Suppl. material 1: figs S3, S4). Nucleotide comparisons with *Cyberlindnera misumaiensis* NRRL Y-17389 (type strain) revealed the following sequence divergences: CGMCC 2.8853 differed by 91 substitutions and 25 indels in the ITS region and 51 substitutions and seven indels in the D1/D2 domain; Y-18-1-13-6 exhibited 94 substitutions and 26 indels in ITS and 53 substitutions and seven indels in D1/D2. The result suggested that the CGMCC 2.8853 clade represented a novel species in the genus *Cyberlindnera*.

### ﻿Taxonomy

#### ﻿ Microbotryozyma lacustris

Taxon classificationFungiMicrobotryalesUstilentylomataceae

L. Tian, Y. Y. Zheng, D. Phurbu & Q. M. Wang sp. nov.

50D39D96-BED0-5ECE-A49A-D11565272D0A

Fungal Names: FN 572954

860640

Fig. 3

##### Etymology.

The species is named after the lake habitat where the type strain was isolated.

##### Holotype.

China • Xizang Autonomous Region, Nyingchi City, Gongbo’gyamda County, Basom Lake, from freshwater, GPS: 30°02'11"N, 93°78'53"E, 3440 m a.s.l., on 15 August 2023, Y. Y. Zheng (holotype CGMCC 2.8854^T^ permanently preserved in a metabolically inactive state, ex-holotype JCM 10420 = ZYY1779).

##### Description.

**Figure 3.**
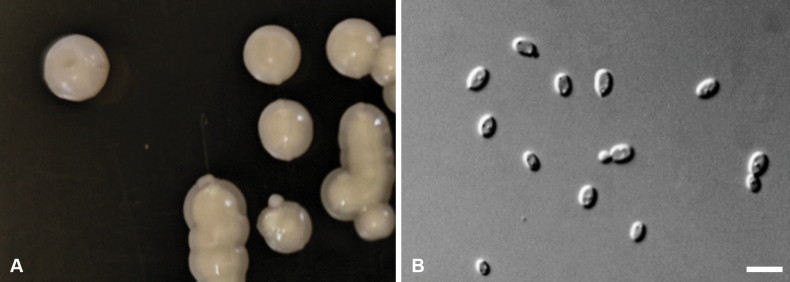
Morphology of *M. lacustris* sp. nov. (strain CGMCC 2.8854^T^). A. Individual colonies by streaking onto Potato Dextrose Agar (PDA) after 3 days; B. Cylindrical arthroconidia on yeast extract–malt extract (YM) after three days of growth at 17 °C. Scale bars: 10 μm.

***Culture characteristics***: After 3 days of incubation in YM broth at 17 °C, cells were ellipsoidal to ovoid, measuring 1.9–3.9 × 3.5–6.5 µm, and reproduced by monopolar budding (Fig. 3). After one month under the same conditions, prominent rings and sediment were present. On YM agar at 17 °C for three days, colonies were creamy, smooth, glossy, and exhibited surface ridges with serrated margins. Pseudohyphae were not formed on cornmeal agar. No ascospores or sexual structures were observed on YM, PDA, V8, or cornmeal agar after six weeks. Ballistoconidia were not produced. ***Physiological and biochemical characteristics***: D-Glucose, sucrose, melibiose, D-arabinose, D-ribose, L-rhamnose, D-mannitol, and raffinose (delayed and weak) were assimilated. The following carbon sources were assimilated weakly or after a delay: D-galactose, sorbose, maltose, cellobiose, trehalose, lactose, melezitose, D-xylose, N-acetyl-D-glucosamine, ethanol, glycerol, galactitol, and hexadecane. Soluble starch, L-arabinose, methanol, erythritol, ribitol, D-glucitol, α-methyl-D-glucoside, DL-lactic acid, succinic acid, citric acid, and inositol were not assimilated. Ammonium sulfate was utilized as a sole nitrogen source; potassium nitrate, sodium nitrite, L-lysine, ethylamine hydrochloride, and cadaverine dihydrochloride were not utilized. Starch-like compounds were not produced. Growth in vitamin-free medium was weak. No growth occurred on 50% (w/w) glucose–yeast extract agar.

##### Materials examined.

China • Xizang Autonomous Region, Nyingchi City, Gongbo’gyamda County, Basom Lake, from freshwater, GPS: 29°98'13"N, 93°86'59"E, 3390 m a.s.l., on 21 July 2025, L. Tian, Y. Y. Zheng, D. Phurbu & Q. M. Wang (living culture BSC-W-3-4, BSC-W-7-4).

##### Notes.

Strains CGMCC 2.8854^T^, BSC-W-3-4, and BSC-W-7-4, identified as *Microbotryozyma lacustris*, cluster within the genus *Microbotryozyma* but are phylogenetically distinct from their closest relatives, *M. collariae* and *M. swertiae*. The D1/D2 domain sequence of strain CGMCC 2.8854 differs by seven substitutions (1.39%) from that of *M. collariae* ATCC MYA-4666^T^, while the ITS region shows 44 mismatches (8.78%, including 29 substitutions and 15 indels), values that exceed thresholds commonly accepted for species delineation in yeasts. Phenotypically, *M. lacustris* can be clearly distinguished from its congeners by its unique carbon and nitrogen assimilation profile. Specifically, it assimilates melibiose, L-rhamnose, galactitol (delayed), and hexadecane (delayed), all of which are not utilized by *M. collariae* or *M. swertiae*. Conversely, it fails to assimilate α-methyl-D-glucoside, potassium nitrate, or ethylamine hydrochloride, compounds that are utilized by both related species. These consistent phenotypic differences, summarized in Table 3, corroborate the phylogenetic data and firmly support its status as a novel species.

**Table 3.** Phenotypic characteristics differentiating *M. lacustris* sp. nov. from its closest relatives, *M. collariae* and *M. swertiae*.

	Characteristic	*M. lacustris*	*M. swertiae*	*M. collariae*
Assimilation of:	Maltose	DW	+	+
	Cellobiose	DW	+	+
	Trehalose	W	+	+
	Lactose	W	+	+
	Melibiose	+	–	–
	D-Xylose	DW	+	–
	D-Arabinose	+	–	+
	D-Ribose	+	DW	–
	L-Rhamnose	+	–	–
	Ethanol	D	–	–
	Ribitol	–	DW	–
	Galactitol	D	–	–
	D-Glucitol	–	+	D
	a-Methyl-D-Glucoside	–	+	+
	Succinic acid	–	DW	N
	Hexadecane	D	–	N
	Vit-free	DW	N	+
	Ammonium sulfate	+	+	W
	Potassium nitrate	–	+	W
	Ethylamine hydrochloride	–	+	W

Note: +, positive; –, negative; w, weakly positive; d, delayed; dw, delayed weak; n, not determined.

#### ﻿ Cyberlindnera basumtsoensis

Taxon classificationFungiSaccharomycetalesPhaffomycetaceae

L. Tian, Y. Y. Zheng, D. Phurbu & Q. M. Wang sp. nov.

DAD99262-53C0-5342-9E16-808DA935371E

Fungal Names: FN 573011

860641

Fig. 4

##### Etymology.

The species is named after the place where the type strain was isolated.

##### Holotype.

China • Xizang Autonomous Region, Nyingchi City, Gongbo’gyamda County, Basom Lake, from freshwater, GPS: 30°02'11"N, 93°78'53"E, 3440 m a.s.l., on 15 August 2023, Y. Y. Zheng, (holotype CGMCC 2.8853^T^ permanently preserved in a metabolically inactive state, ex-holotype JCM 10419 = ZYY005).

##### Description.

**Figure 4.**
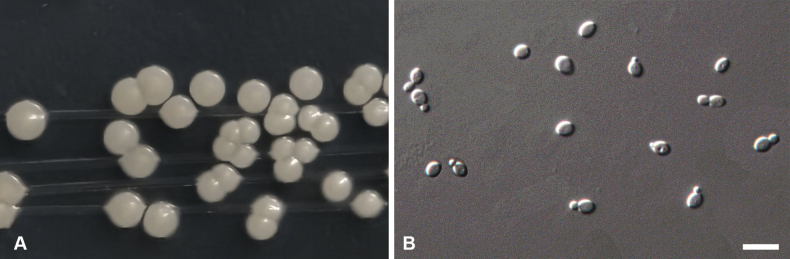
Morphology of *C. basumtsoensis* sp. nov. (strain CGMCC 2.8853^T^). A. Individual colonies by streaking onto Potato Dextrose Agar (PDA) after 3 days; B. Cylindrical arthroconidia on yeast extract–malt extract (YM) after three days of growth at 17 °C. Scale bars: 10 μm.

***Culture characteristics***: After 3 days of incubation in YM broth at 17 °C, cells were ellipsoidal to ovoid, measuring 2.6–3.7 × 3.0–5.5 µm, and reproduced by monopolar budding (Fig. 4). After one month under the same conditions, conspicuous rings and sediment were present. On YM agar at 17 °C for 3 days, colonies were creamy-white, butyrous, and emitted a characteristic aroma; the center was slightly raised and produced filaments when lifted with a loop. Pseudohyphae were not formed on cornmeal agar. No ascospores or sexual structures were observed on YM, PDA, V8, or cornmeal agar after 6 weeks. Ballistoconidia were not produced. ***Physiological and biochemical characteristics***: D-Glucose, sucrose, melibiose, raffinose, melezitose, inulin, soluble starch, L-rhamnose, ethanol, glycerol, galactitol, D-mannitol, D-glucitol, DL-lactic acid, and succinic acid were assimilated. D-Galactose, maltose, cellobiose, and citric acid were assimilated weakly or after a delay. L-Sorbose, trehalose, lactose, L-arabinose, D-arabinose, D-ribose, N-acetyl-D-glucosamine, methanol, erythritol, ribitol, α-methyl-D-glucoside, inositol, and hexadecane were not assimilated. Cadaverine dihydrochloride, L-lysine (weakly), and potassium nitrate (weakly) were utilized as sole nitrogen sources; ammonium sulfate, sodium nitrite, and ethylamine hydrochloride were not utilized. Starch-like compounds were not produced. Growth in vitamin-free medium was weak. No growth occurred on 50% (w/w) glucose–yeast extract agar.

##### Materials examined.

China • Qinghai Province, Golog Tibetan Autonomous Prefecture, Jiuzhi County, County Road X740, isolated from a plant, GPS: 33°62'81"N, 101°54'60"E, 3525.7 m a.s.l., on 18 September 2025 (living culture Y-18-1-13-6).

##### Notes.

Strains CGMCC 2.8853^T^ and Y-18-1-13-6 represent a novel species within the genus *Cyberlindnera*, for which we propose the name *C. basumtsoensis*. The type strain CGMCC 2.8853 showed significant genetic divergence from its closest phylogenetic neighbors, with sequence disparities of 10.22% in the D1/D2 domain and 15.66% in the ITS region, unequivocally supporting its status as a distinct species. Phenotypically, *C. basumtsoensis* exhibits a distinctive combination of traits, including weak assimilation of D-galactose, delayed assimilation of raffinose and inulin, weak utilization of potassium nitrate and L-lysine, and an inability to assimilate trehalose or ethylamine hydrochloride (Table 4). These characteristics provide a clear phenotypic signature that differentiates it from other described *Cyberlindnera* species.

**Table 4.** Phenotypic characteristics differentiating *C. basumtsoensis* sp. nov. from its closest relatives, *C. xishuangbannaensis* and *C. sylvatica*.

	Characteristic	*C. basumtsoensis*	*C. xishuangbannaensis*	*C. sylvatica*
Fermentation	Glucose	+	+	+
	Sucrose	–	+	W
	inulin	N	W	N
Assimilation of:	D-Galactose	W	–	W
	Cellobiose	D	+	+
	Trehalose	–	+	+
	Melibiose	W	N	N
	Raffinose	D	+	–
	Melezitose	D	N	N
	Inulin	D	+	–
	Soluble starch	+	W	+
	L-Arabinose	–	W	N
	D-Arabinose	–	N	W
	D-Ribose	–	W	W
	L-Rhamnose	+	+	+
	Ribitol	–	W	–
	Galactitol	+	–	–
	a-Methyl-D-Glucoside	–	+	+
	Citric acid	W	+	+
	Vit-free	W	N	–
	Potassium nitrate	W	–	–
	L-lysine	W	+	+
	Ethylamine hydrochloride	–	–	+
	Cadaverine dihydrochloride	+	–	N

Note: +, positive; –, negative; w, weakly positive; d, delayed; dw, delayed weak; n, not determined.

## ﻿Discussion

This study describes the isolation and characterization of two novel yeast species, *Microbotryozyma lacustris* sp. nov. and *Cyberlindnera basumtsoensis* sp. nov., from the freshwater environment of Basom Lake in Tibet. These discoveries not only enrich the species diversity within their respective genera but, more significantly, expand our understanding of their ecological adaptability.

The genus *Microbotryozyma* was previously represented by species predominantly isolated from terrestrial environments (Wurzbacher et al. 2010). The discovery of *M. lacustris* provides the first clear evidence of a representative from a freshwater habitat. This finding corroborates recent studies that have detected members of this genus in other Tibetan lakes and Japanese freshwater environments, collectively revealing a previously underappreciated distribution pattern of *Microbotryozyma* in freshwater ecosystems (Urano et al. 2019; Hao et al. 2021). Our phylogenetic analyses indicate that *Microbotryozyma* forms a tight complex with the genus *Aurantiosporium*, suggesting that the current morphology-based taxonomic framework may require revision incorporating molecular phylogenetic data. Despite these higher-level taxonomic considerations, the clear phylogenetic affiliation of our strain with the type species of *Microbotryozyma*, supported by stable morphological characteristics, firmly justifies its placement within this genus.

The genus *Cyberlindnera* is renowned for its ecological versatility and biotechnological potential, particularly in lignocellulose degradation and xylose metabolism (Barros et al. 2021; Bonthong et al. 2025). The isolation of *C. basumtsoensis* from a pristine high-altitude lake significantly expands the known ecological range of this genus and underscores its adaptability to oligotrophic freshwater environments. While our physiological data confirm that *C. basumtsoensis* shares the typical ability of the genus to assimilate a wide range of carbon sources, its specific profile—including the delayed assimilation of raffinose and inulin and weak growth on vitamin-free medium—may represent a unique adaptation to its native habitat. This phenotypic distinctiveness, coupled with its significant genetic divergence from known species, suggests the evolution of a novel ecotype within the genus.

The recovery of *C. basumtsoensis* from Basum Tso aligns with previous reports of *Cyberlindnera* species participating in the decomposition of organic matter in various ecosystems (Soto-Robles et al. 2019). Its presence in this isolated lake suggests a potential, yet unconfirmed, role in aquatic carbon cycling. Furthermore, the strain’s ability to assimilate compounds like soluble starch and its weak utilization of nitrate hint at metabolic capabilities that merit further investigation. Given the documented potential of congeners like *C. jadinii* and *C. fabianii* in biotechnology (Sousa-Silva et al. 2021; Bonthong et al. 2025), the unique origin and physiological traits of *C. basumtsoensis* position it as a promising candidate for exploring novel enzymes or metabolic pathways, potentially unlocking new applications in the conversion of agricultural residues or other biotechnological processes.

In conclusion, through the characterization of these two novel yeast species, our study reveals the presence and adaptation of *Microbotryozyma* and *Cyberlindnera* in freshwater ecosystems, particularly in the unique niche of a high-altitude lake. These findings underscore the importance of extreme or specialized environments as hotspots for microbial diversity exploration and lay the groundwork for future research into the ecological functions, evolutionary adaptations, and potential applications of these yeast lineages.

## Supplementary Material

XML Treatment for Microbotryozyma lacustris

XML Treatment for Cyberlindnera basumtsoensis

## ﻿Additional information

### Use of AI

No use of AI was reported.

### Author ORCIDs

Lin Tian ￼ https://orcid.org/0009-0001-8204-4802

Yan-Yan Zheng ￼ https://orcid.org/0000-0003-3405-8932

### Data availability

All of the data that support the findings of this study are available in the main text or Supplementary Information.

## Supplementary materials

Supplementary material 1 Phylogenetic trees Data type doc https://binary.pensoft.net/file/1492691This dataset is made available under the Open Database License (http://opendatacommons.org/licenses/odbl/1.0/). The Open Database License (ODbL) is a license agreement intended to allow users to freely share, modify, and use this Dataset while maintaining this same freedom for others, provided that the original source and author(s) are credited.Lin Tian, Dorji Phurbu, Yan-Yan Zheng
