# Supplementary material for: ﻿Microbotryozyma lacustris sp. nov. (Basidiomycota, Ustilentylomataceae) and Cyberlindnera basumtsoensis sp. nov. (Ascomycota, Phaffomycetaceae), two novel yeasts isolated from freshwater Lake Basom Tso, China
Source: MycoKeys. 2025 Dec 15;126:135–50. doi: 10.3897/mycokeys.126.173807 (PMC12723391; doi:10.3897/mycokeys.126.173807)
Supplement: Supplementary material 1 — Phylogenetic trees [file mycokeys-126-135-s001.doc]

***Microbotryozyma lacustris* sp. nov. (*Basidiomycota*, *Ustilentylomataceae*) and *Cyberlindnera basumtsoensis* sp. nov. (*Ascomycota*, *Phaffomycetaceae*), two novel yeasts isolated from freshwater Lake Basom Tso, China**

Lin Tian^1^, Dorji Phurbu^1^, Yan-Yan Zheng^1*^

^1^Tibet Plateau Institute of Biology, Lhasa 850000, China

****Correspondence*:** Yan-Yan Zheng, sws_zhs@sti.xizang.gov.cn

**Running title:** *Microbotryozyma lacustris* sp. nov. and *Cyberlindnera basumtsoensis* sp. nov.

**Subject category**: New taxa-Eukaryotic Microorganisms

The GenBank accession numbers for the 26S rRNA gene D1/D2 domain and the ITS region of strain CGMCC 2.8854^T^ are PX048001 and PX048003, respectively, while those of strain CGMCC 2.8853^T^ are PX048002 and PX048004, respectively. The Fungal Names registration numbers are FN 572954 for *Microbotryozyma lacustris* sp. nov. and FN 573011 for *Cyberlindnera basumtsoensis* sp. nov. The MycoBank numbers are MB860640 and MB860641, respectively.


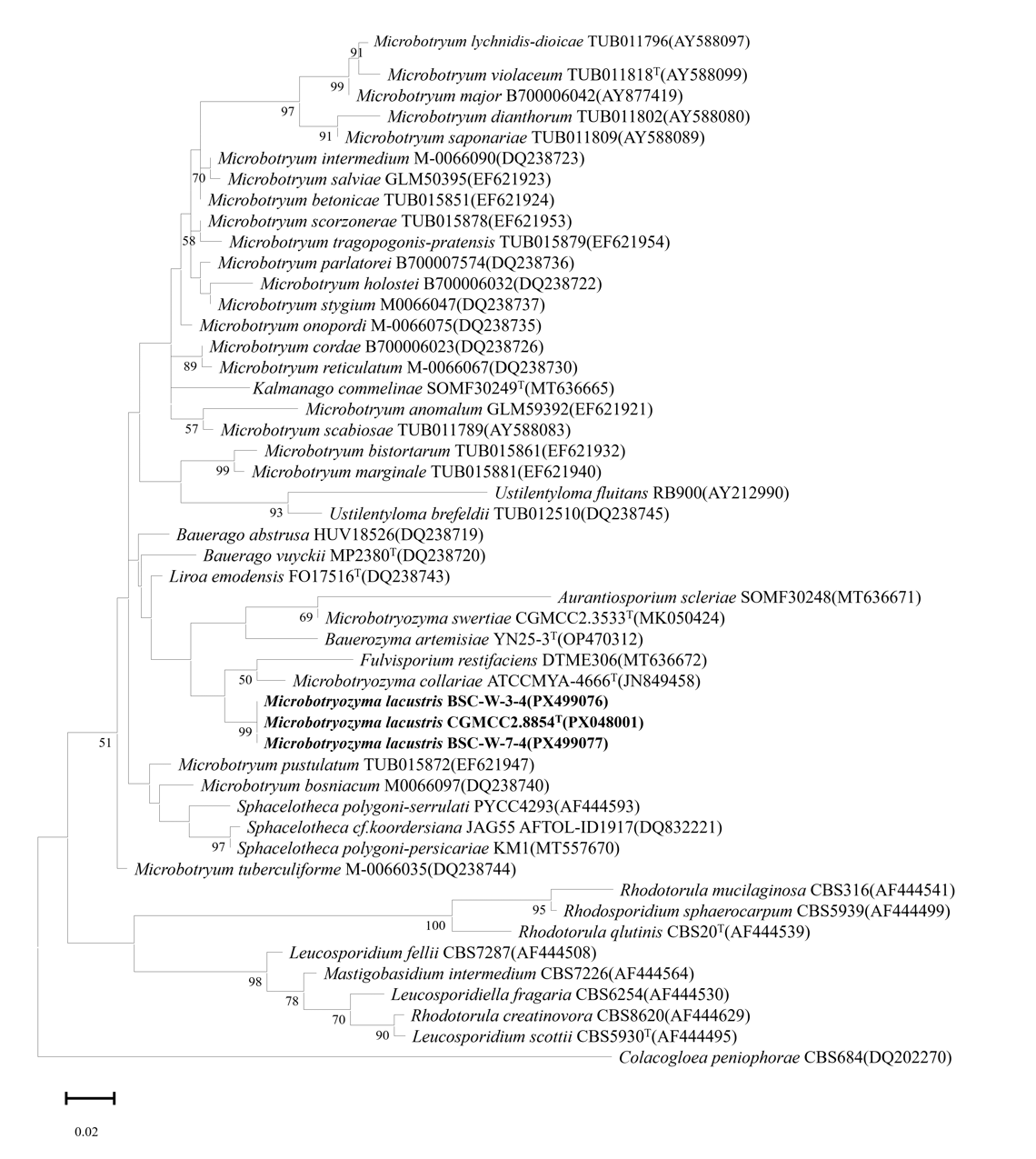


Fig S1 Phylogenetic tree constructed from the combined sequences of the ITS regions, showing the phylogenetic positions of the type strain CGMCC 2.8854ᵀ and related species. Reference strains included in the tree were either type strains of closely related species within the genus or strains widely cited in previous studies to ensure accurate phylogenetic comparison. Maximum likelihood bootstrap values (ML-BS ≥ 70%) are shown above the branches. *Colacogloea peniophorae* CBS 684ᵀ (accession numbers: DQ202270) was used as the outgroup. The scale bar represents a patristic distance of 0.02.


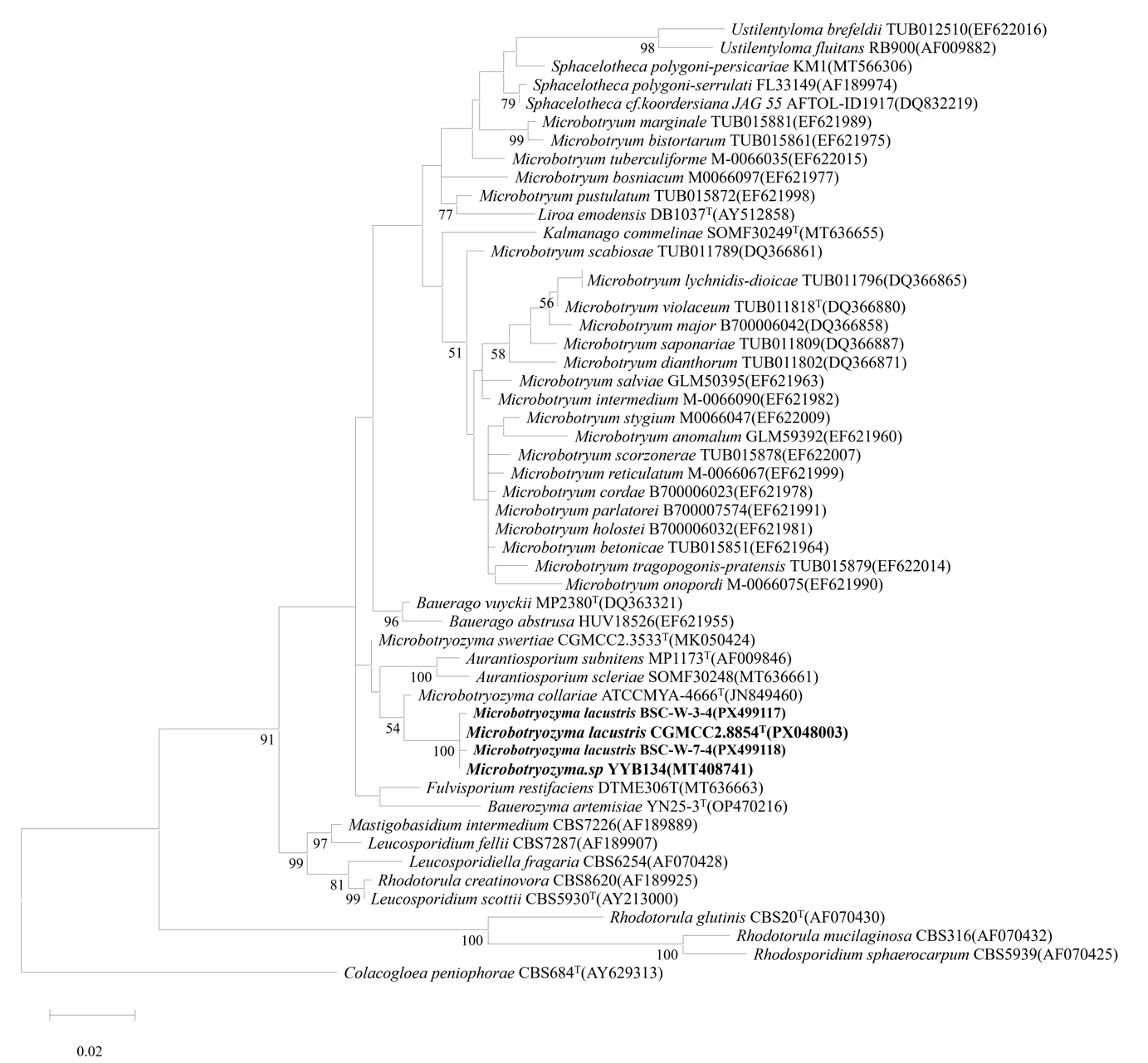


Fig S2 Phylogenetic tree constructed from the combined sequences of the D1/D2 regions, showing the phylogenetic positions of the type strain CGMCC 2.8854ᵀ and related species. Reference strains included in the tree were either type strains of closely related species within the genus or strains widely cited in previous studies to ensure accurate phylogenetic comparison. Maximum likelihood bootstrap values (ML-BS ≥ 70%) are shown above the branches. *Colacogloea peniophorae* CBS 684ᵀ (accession numbers: AY629313) was used as the outgroup. The scale bar represents a patristic distance of 0.02.


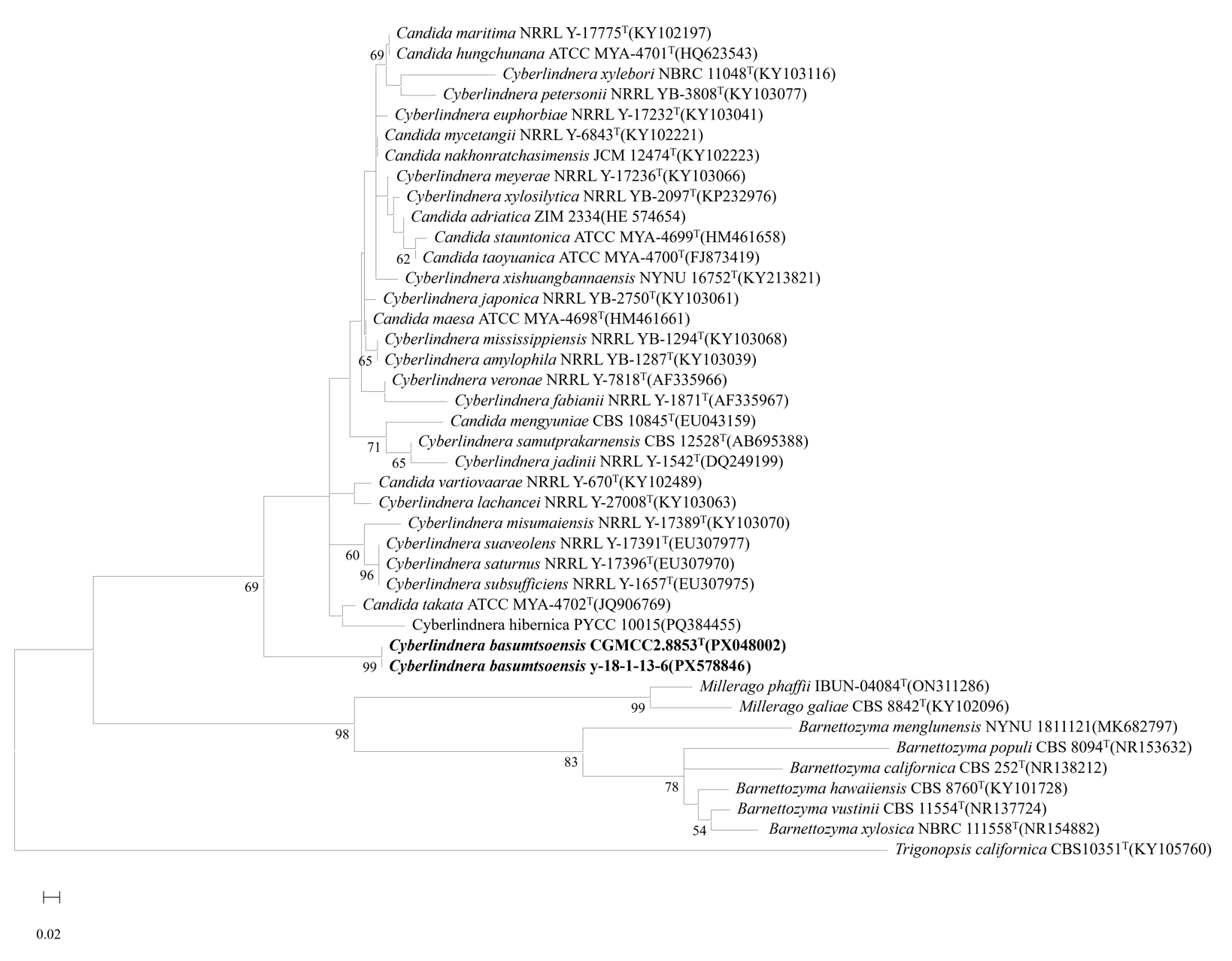


Fig S3 Phylogenetic tree constructed from the combined sequences of the ITS regions, showing the phylogenetic positions of the type strain CGMCC 2.8853ᵀ and related species. Reference strains included in the tree were either type strains of closely related species within the genus or strains widely cited in previous studies. Maximum likelihood bootstrap values (ML-BS ≥ 50%) are shown above the branches. *Trigonopsis californica* CBS 10351 (KY105760) was used as the outgroup. Bar, patristic distance of 0.02.


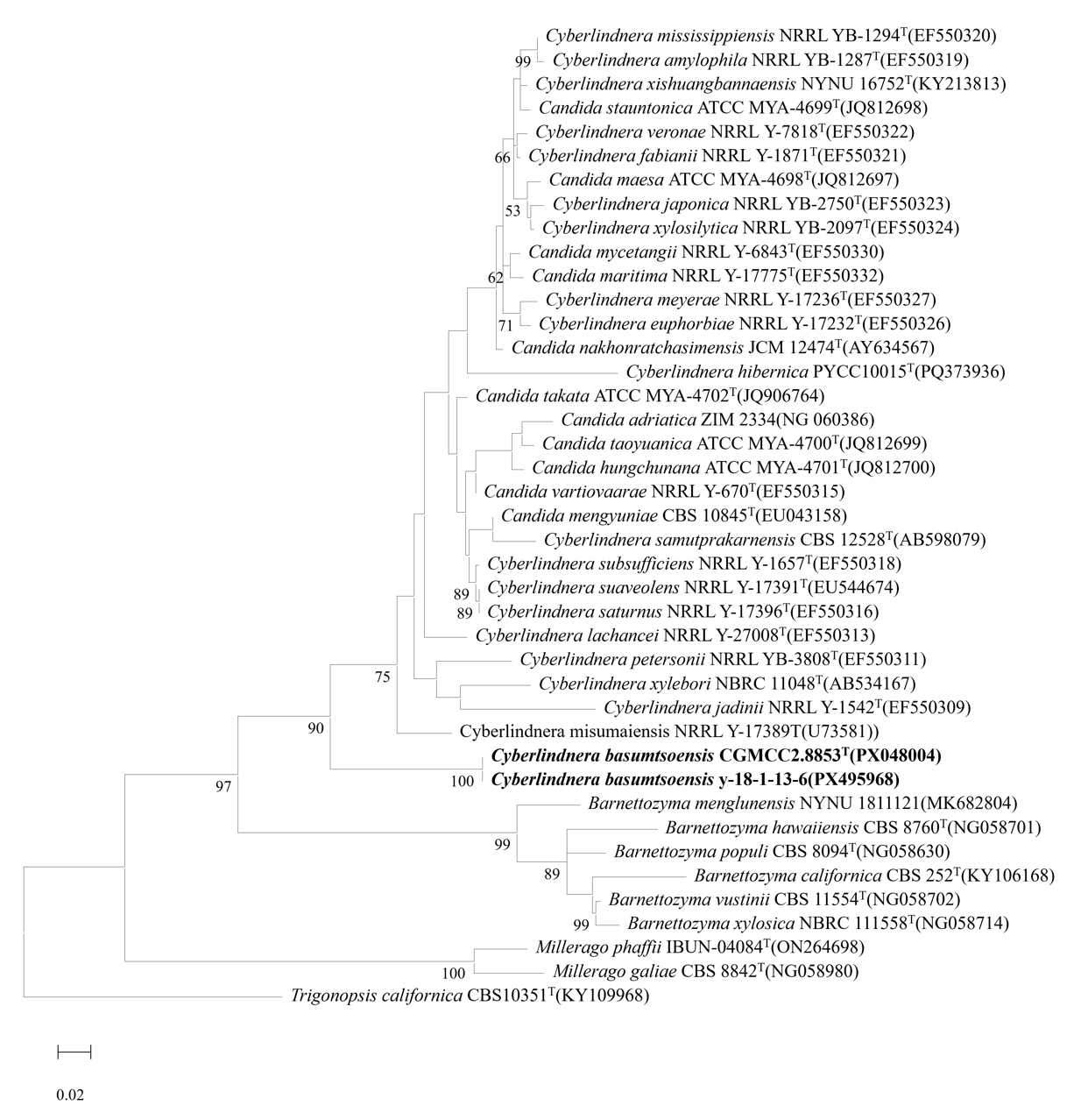


Fig S4 Phylogenetic tree constructed from the combined sequences of the D1/D2 regions, showing the phylogenetic positions of the type strain CGMCC 2.8853ᵀ and related species. Reference strains included in the tree were either type strains of closely related species within the genus or strains widely cited in previous studies. Maximum likelihood bootstrap values (ML-BS ≥ 50%) are shown above the branches. *Trigonopsis californica* CBS 10351 (KY109968) was used as the outgroup. Bar, patristic distance of 0.02.
